# Supplementary material for: Effect of high and low risk susceptibles in the transmission dynamics of COVID-19 and control strategies
Source: PLoS One. 2021 Sep 15;16(9):e0257354. doi: 10.1371/journal.pone.0257354 (PMC8443082; doi:10.1371/journal.pone.0257354)
Supplement: Attachment — Submitted filename: Review-PONE-D-21-22554.pdf [file pone.0257354.s001.pdf]

Referee's report of the article # PONE-D-21-22554

In the note, titled:"**Effect of High and Low Risk Susceptible in the Transmission Dynamics of COVID-19 and Control Strategies**", the authors formulated and analyzed a deterministic model for the transmission of COVID-19 and evaluated control strategies for the epidemic by considering two susceptible classes, a high risk and a low risk group.

After describing the model, the authors derived some basic properties using standard dynamical systems theory. The system has two steady states, a disease free equilibrium, when the disease dies out in the long run and an endemic equilibrium, where the disease is endemic in the population.

The work is characterized by modernity, and the model presented in the paper is new and serves a vital topic that our world suffers from these days, which is the emerging corona virus (COVID-19).

I would be grateful to recommend this paper for publication in PLOS ONE.
